# Supplementary material for: Deep Characterization and Comparison of Different Retrovirus-like Particles Preloaded with CRISPR/Cas9 RNPs
Source: Int J Mol Sci. 2023 Jul 13;24(14):11399. doi: 10.3390/ijms241411399 (PMC10380221; doi:10.3390/ijms241411399)
Supplement: Supplementary file 1 [file ijms-24-11399-s001.zip › ijms-2486490-supplementary.pdf]

SUPPLEMENTARY DATA

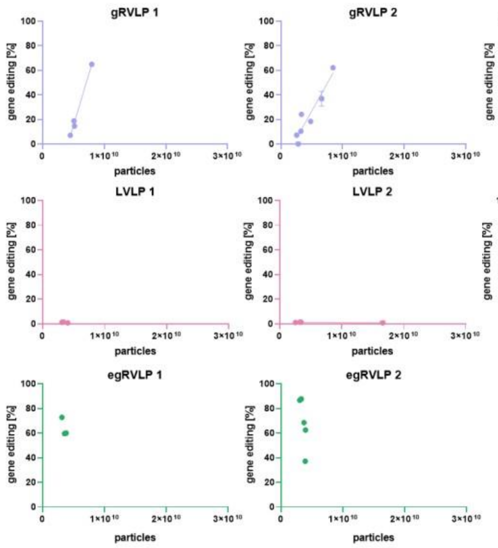

Supplementary Figure S1: Correlation of particle numbers and GE efficiency. GE efficiency achieved with VSV-G-pseudotyped GFP-programmed RVLPs in a 293T-derived reporter cell line produced with different amounts of plasmids plotted against the number of particles used for transduction. Particle concentrations were measured using NTA. Vertical error bars represent ± SE, horizontal error bars represent ± SD, n ≥ 3. For egRVLPs, individual values of particle numbers from different preparations were too close to calculate meaningful correlation (compare also Fig. 2C).

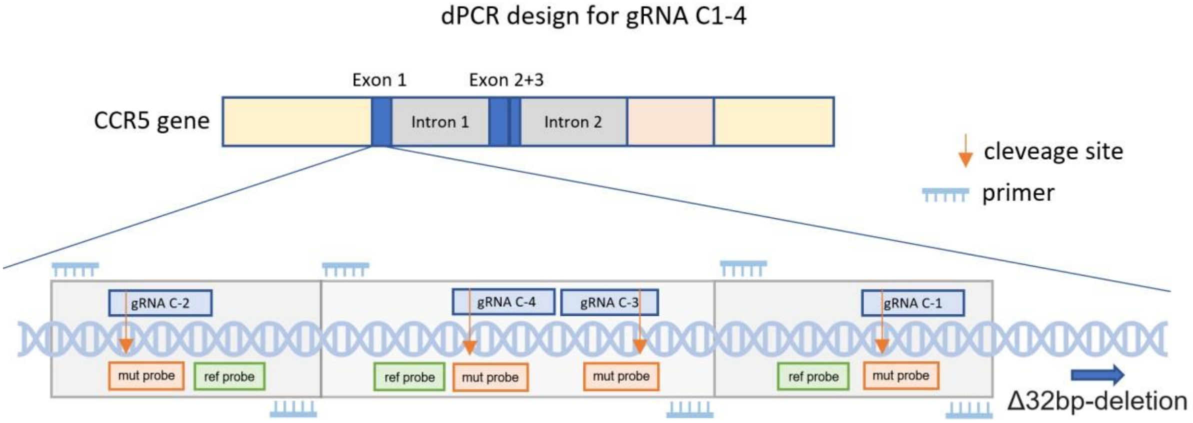

Supplementary Figure S2: Design of the GEF-dPCR reaction. Schematic representation of the target sites of C-1-C-4 programmed CRISPR/Cas9 within the CCR5 gene and the binding sites of primers, mutation probes, and reference probes for the GEF-dPCR.

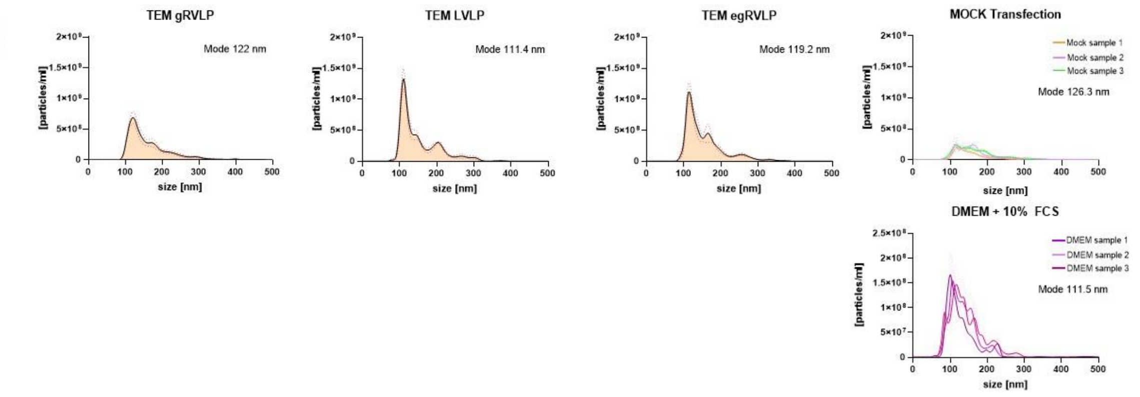

Supplementary Figure S3: Morphological Analysis of VLP by Nanoparticle tracking analysis (NTA). NTA was applied to measure particle size distribution and mode sizes for aliquots of the different RVLP samples as indicated, for cell culture supernatant from cells undergoing standard transfection without DNA (“mock”, n=3), and for the used cell culture medium (n=3). The dotted line represents ± SE

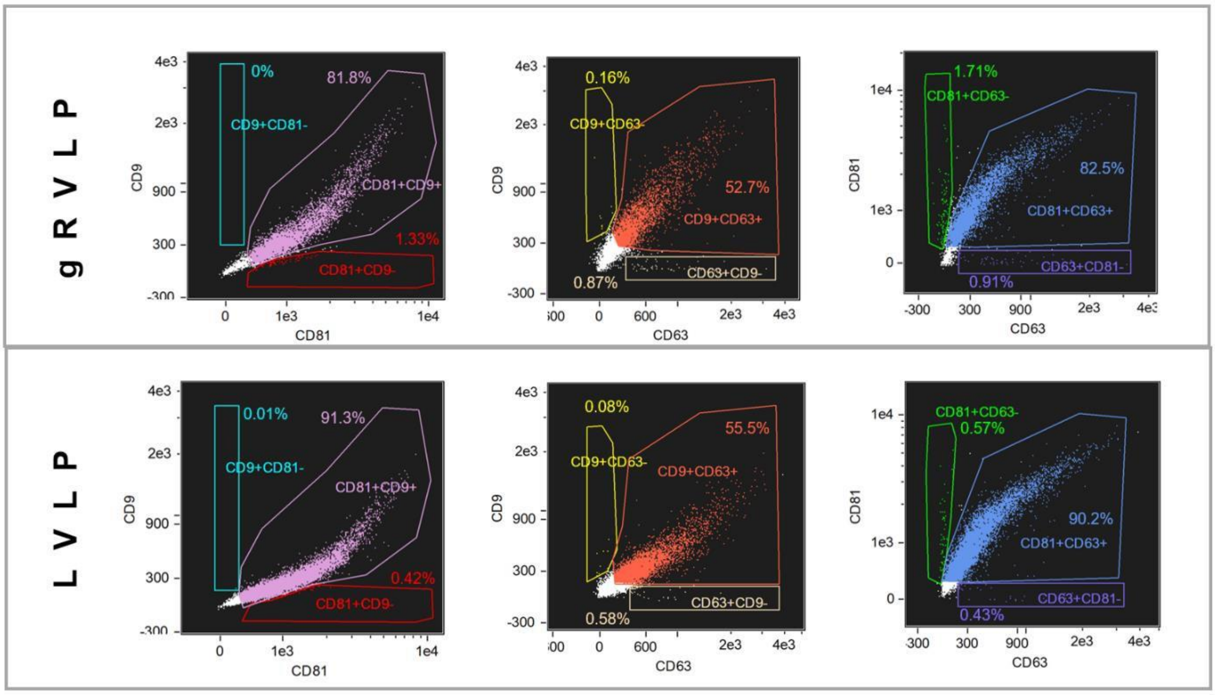

Supplementary Figure S4: Gating strategy of IFC analysis. Image flow cytometry analysis of gRVLPs and LVLPs stained with anti-CD9-PE, anti-CD81-FITC and anti-CD63-PacBlue allows discrimination of subpopulations differing in their tetraspanin composition. Data is presented as complete dot plots.

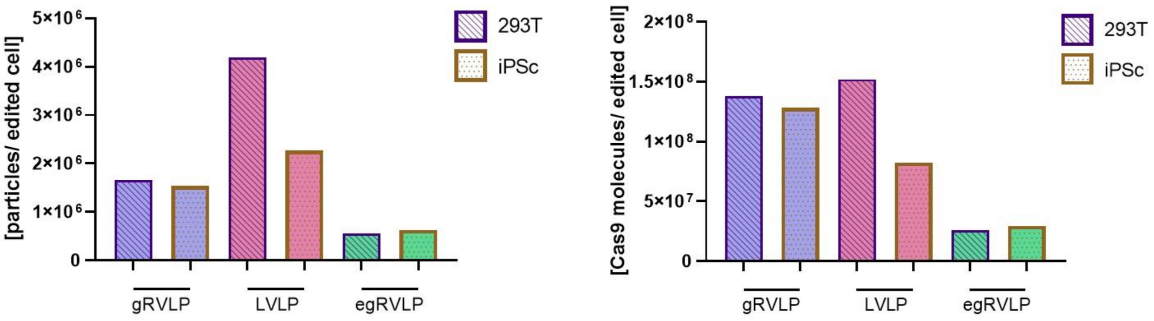

Supplementary Figure S5: Particles and Cas9 molecules used per edited cell in knockout experiments. An analysis of the efficiency of the presented RVLP systems, addressing the number of particles/Cas9 molecules needed to edit 1 cell. The y-axis shows the particles (left bar graph) and the amount of Cas9 molecules (right bar graph) used for transduction divided by the number of edited cells (number of GFP-/CCR5- cells in the cell culture). The analysis includes knockout experiments in 293T cells and iPS cells. Data are represented as mean ± SD, n = 1.

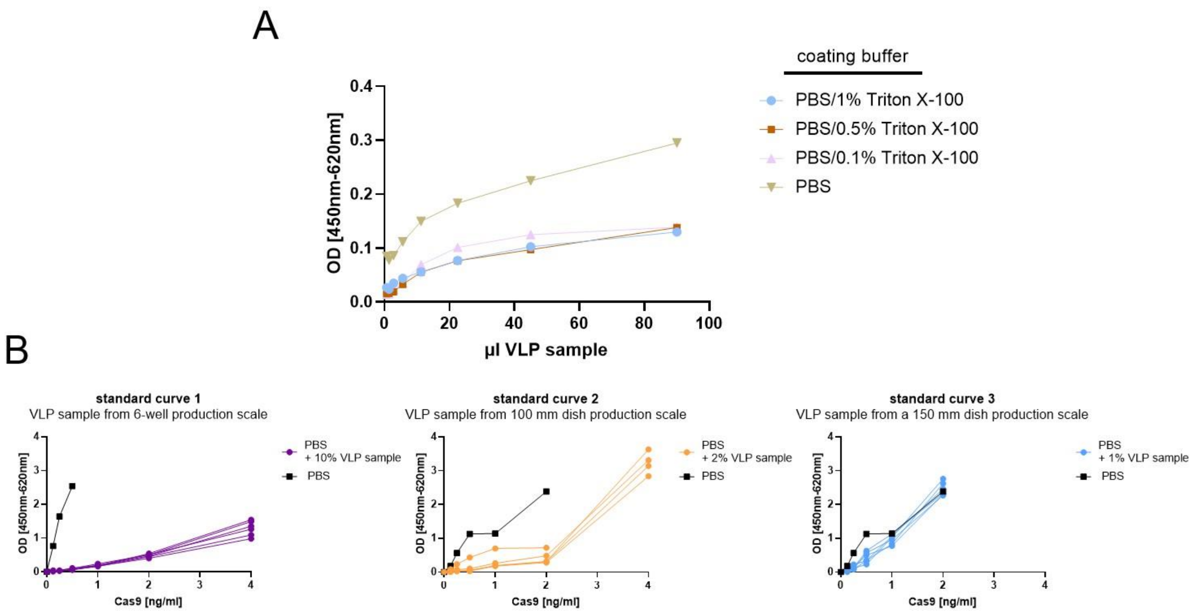

Supplementary Figure S6: Optimization steps and generation of standard curves for indirect ELISA. (A) Optical density (OD) values obtained from the same VLP sample, coated using PBS + (0.1%, 0.5%, or 1%) Triton X-100 or PBS only as the coating buffer. **(B)** Comparison of OD values from ELISA measurements of recombinant Cas9 diluted in either PBS or PBS/ 10%, 2%, or 1% VLP sample allows quantification of the inhibition of the OD Values. When using defined percentages (10%, 2%, 1%) of VLP samples from defined scales of production (6-well, 100 mm dish, 150 mm dish), the inhibition of signal is consistent. For each standard curve gRVLP, LVLP and egRVLP were used at least once, n ≥ 5. OD values that are above the detection limit are not displayed.

Primer sequences

		Sequence
CCR5 -1 (C-1)	Forward	5' ACCGGCAGCATAGTGAGCCCAGA 3'
	Reverse	5' AACTCTGGGCTCACTATGCTGCCGCC 3'
CCR5-2 (C-2)	Forward	5' ACCGTAATAATTGATGTCATAGAT 3'
	Reverse	5' AACATCTATGACATCAATTATTATAC 3'
CCR5-3 (C-3)	Forward	5' ACCGTCATCCTGATAAACTGCAAA 3'
	Reverse	5' AACTTTGCAGTTTATCAGGATGAG 3'
CCR5-4 (C-4)	Forward	5' ACCGTCAGCCTTTTGCAGTTTATC 3'
	Reverse	5' AACGATAAACTGCAAAAGGCTGAA 3'
CCR5 – del1 (C-1del)	Forward	5' ACCGGGCAGCATAGGAGCCCAGA 3'
	Reverse	5' AACTCTGGGCTCCTATGCTGCCG 3'
GFP	Forward	5' ACCGAGCTGGACGGCGACGTAAA 3'
	Reverse	5' AACTTTACGTCGCCGTCCAGCTC 3'

Supplementary table S1. Sequences of primers used for molecular cloning into the cloning site of LeGO-iC2 to generate gRNA-expressing plasmids.

	gRVLP		egRVLP		LVLP	
gRNA-expressing plasmid	LeGO-iC2 (addgene #27345)	10 µg	LeGO-iC2 (addgene #27345)	10 µg	LeGO-iC2 (addgene #27345)	10 µg
GagPol	pcDNA3.MLVgp	5.6 µg	pcDNA3.MLVgp	5.6 µg	pMDLg/pRRE	9 µg
GagCas9	BIC-Gag-Cas9 (addgene #119942)	19 µg	pCMV-MMLVgag -3xNES-Cas9 (addgene #181752)	19 µg	pJRH029 (addgene #171060)	19 µg
VSV-G	phCMV-VSV-G	1 µg	phCMV-VSV-G	1 µg	phCMV-VSV-G	1 µg

Supplementary table S2. gRNA, GagPol, GagCas9, and VSV-G expressing plasmids used for transfection of 293T cells to produce RVLPs. The amounts of plasmid are adjusted for production in 100-mm cell-culture dishes and applied unless explicitly described otherwise.

Primer and probes used in GEF-dPCR experiment

gRNA		Sequence
C-1/ C-1del	fw	5' CAAAAGGCTGAAGAGCATGACTG 3'
	Rev	5' AGAGCCCTGTCAAGAGTTGACAC 3'
	ref	5' [HEX] TACCTGCTCAACCTGGCCATCTCTGAC[BHQ1] 3'
	mut	5' [FAM]CTTCTGGGCTCACTATGCTGCCGC[BHQ1] 3'
C-2	fw	5' TTTATGCACAGGGTGGAACAAG 3'
	Rev	5' AGAGCCCTGTCAAGAGTTGACAC 3'
	ref	5' [HEX]TGTTCATCTTTGGTTTTGTGGGCAAC[BHQ1] 3'
	mut	5' [FAM]TGGATTATCAAGTGTCAAGTCCAATCTATGACATCAA[BHQ1] 3'
C-3	fw	5' CTGCCTCCGCTCTACTCACT 3'
	Rev	5' CCCAGAAGGGGACAGTAAGA 3'
	ref	5' [HEX]CTTTGGTTTTGTGGGCAACATGCT[BHQ1] 3'
	mut	5' [FAM]CTGCAAAAGGCTGAAGAGCATGACT[BHQ1] 3'
C-4	fw	5' CTGCCTCCGCTCTACTCACT 3'
	Rev	5' CCCAGAAGGGGACAGTAAGA 3'
	ref	5' [HEX]CTTTGGTTTTGTGGGCAACATGCT[BHQ1] 3'
	mut	5' [FAM]ATAAACTGCAAAAGGCTGAAGAGCATGAC[BHQ1] 3'

Supplementary table S3. Primers and probes were designed following the protocol provided by Mock et al. 2016. Probes are either marked with HEX or FAM as fluorophores with BHQ1 added for signal quenching.

Reference:

Mock U, Hauber I, Fehse B (2016) Digital PCR to assess gene-editing frequencies mediated by designer nucleases. *Nature Protocols* **11**, 598–615.
